# Performance of a Sequencing Biofilter Coupled with a Dual-Media Granular Activated Carbon Filter for PFAS Mitigation in Landfill Leachate

**DOI:** 10.3390/molecules31111788

**Published:** 2026-05-22

**Authors:** Flor Ximena Cadena-Aponte, Sofiane El Barkaoui, Patricia Plaza-Bolaños, Ana Agüera, Rossella Annelio, Cristina De Ceglie, Subhoshmita Mondal, Giuseppe Bagnuolo, Giuseppe Mascolo, Claudio Di Iaconi

**Affiliations:** 1 Department of Chemistry and Physics, University of Almería, 04120 Almería, Spain; 2 Solar Energy Research Centre (CIESOL), Joint Centre University of Almeria-CIEMAT, Carretera de Sacramento s/n, 04120 Almeria, Spain; 3 Water Research Institute, National Research Council (C.N.R.), Viale F. De Blasio 5, 70123 Bari, Italy; 4 Department of Civil, Environmental, Land, and Construction Engineering and Chemistry, Polytechnic University of Bari, Via E. Orabona n.4, 70125 Bari, Italy

**Keywords:** hybrid treatment system, PFAS breakthrough behavior, short-chain PFAS, long-chain PFAS, mass balance, bed volume

## Abstract

The performance of a sequencing batch biofilter granular reactor (SBBGR), followed by a dual media granular activated carbon (GAC) column, was evaluated in terms of its ability to remove selected per- and polyfluoroalkyl substances (PFAS) from landfill leachate. The results show that the SBBGR achieved an overall reduction of 51%, with the preferential removal of long-chain PFAS, while short-chain PFAS were only partially removed. Subsequent GAC treatment exhibited compound-specific breakthrough behavior, which was governed by chain length. Short-chain PFAS (e.g., perfluorobutanoic acid) exhibited rapid bed volumes at 50% breakthrough (BV_50_ ≈ 88), whereas long-chain PFAS (e.g., perfluorooctanoic acid and perfluorooctanesulfonic acid) were substantially more retained (BV_50_ ≈ 446 and 361, respectively), with perfluorohexanesulfonic acid and perfluorodecanoic acid failing to reach BV_50_ within the monitored period. Mass balance analysis showed that the hybrid GAC column captured ~73% of the influent PFAS mass. This resulted in >80–95% retention of long-chain PFAS and <40% retention of short-chain PFAS. Although long-chain PFAS were preferentially adsorbed, mobile short-chain species dominated residual effluent loads. These findings highlight the need for optimized contact times or dual-media strategies to control the breakthrough of short-chain PFAS.

## 1. Introduction

Per- and polyfluoroalkyl substances (PFAS) constitute a broad class of synthetic organic chemicals that have been widely used since the mid-20th century in a variety of industrial processes and consumer goods [1,2]. These include firefighting foams (notably aqueous film-forming foams, or AFFFs), non-stick cookware, stain-resistant fabrics, waterproof clothing, and food packaging materials [3,4]. The exceptional strength of the carbon–fluorine bond renders PFAS highly resistant to heat, water, and chemical degradation, underpinning both their industrial utility and their extreme persistence in natural environments [5]. Consequently, they have become globally pervasive pollutants, detected in surface and groundwater, soil, sediments, air, and even remote ecosystems such as the Arctic [6]. Their strong bioaccumulative properties have led to widespread detection in wildlife and human biomonitoring studies, indicating long-term internal exposure across populations [7]. Chronic exposure to certain PFAS has been associated with a range of adverse health outcomes, including immunotoxicity, endocrine disruption, liver damage, reproductive impairments, and increased cancer risk [8]. Due to their extreme persistence and mobility, PFAS are now widely referred to as “forever chemicals”, underscoring the challenges associated with their removal using conventional treatment technologies [4].

Despite increasing regulatory efforts, including measures under the Stockholm Convention that have targeted legacy compounds such as perfluorooctanesulfonate (PFOS) and perfluorooctanoic acid (PFOA), thousands of other PFAS remain unregulated or insufficiently characterized [9]. As a result, PFAS contamination has emerged as a truly global environmental concern, with legacy compounds frequently detected in drinking water supplies near industrial and firefighting foam application sites, sometimes exceeding regulatory guidance values [10]. Human biomonitoring studies further confirm widespread internal exposure, with PFAS concentrations in serum and tap water often correlated with regional industrial activity and water source characteristics [11].

The PFAS treatment from aqueous environments remains a considerable technical challenge due to their high solubility and resistance to degradation [12]. Conventional wastewater treatment processes, such as coagulation, filtration, and biological treatment, have generally been ineffective at removing PFAS, highlighting the need for alternative or integrated treatment strategies [13].

Among proposed treatment strategies, the Sequencing Batch Biofilter Granular Reactor (SBBGR) has been investigated as a promising biological system for treating complex, recalcitrant wastewater containing PFAS [12]. By combining the operational flexibility of sequencing batch reactors with granular biomass systems, SBBGR promotes the formation of dense microbial aggregates that can enhance contaminant partitioning and matrix conditioning [14]. The SBBGR functions as a biologically active treatment unit in which a mixed microbial consortium forms a biofilm on the granular support media, facilitating the biodegradation of conventional organic pollutants (e.g., dissolved organic carbon, ammonia) and thereby improving overall water quality [15,16]. In addition, SBBGR systems have been extensively validated for treating recalcitrant industrial wastewater, demonstrating high operational stability and resilience under variable loading conditions [17,18]. Recently, some studies have shown that SBBGR-based processes can significantly attenuate selected PFAS species during the treatment of landfill leachates, particularly long-chain compounds, while achieving limited degradation of short-chain compounds (e.g., perfluorobutanesulfonic (PFBS), perfluorooctanesulfonic (PFOS)) [12,19]. For example, El Barkaoui et al. [19] obtained an overall removal of total PFAS 32% using SBBGR, completely eliminating long-chain PFAS compounds due to the specific properties of the biomass, outperforming conventional biological treatments, while it was ineffective in removing short-chain PFAS. Similarly, De Sanctis et al. [12] reported that the SBBGR system demonstrated high and progressively improving removal of PFAS from landfill leachate, achieving up to 94% total PFAS removal at the highest influent concentrations, in particular long-chain PFAS, such as perfluorooctanoic acid (PFOA) and PFOS, were preferentially removed, while short-chain compounds were less effectively eliminated. These findings highlight the need for more targeted or hybrid treatment approaches to improve the removal efficiency of PFAS compounds, especially short-chain compounds.

One of the proposed solutions to achieve effective PFAS control is the adsorption-based treatment steps, using granular activated carbon (GAC). The latter is among the most applied and practical technologies for PFAS removal from water due to its high specific surface area, well-developed porous structure, and strong affinity for hydrophobic organic compounds [20,21]. It is well known that the PFAS adsorption onto activated carbon is primarily governed by hydrophobic interactions and electrostatic forces, which favor the removal of long-chain PFAS, while shorter-chain and more polar compounds typically exhibit lower adsorption affinity and faster breakthrough [20]. Although PFASs are generally considered recalcitrant to biodegradation, biological treatment within the SBBGR may contribute to PFAS removal. In fact, the very long retention times of SBBGR biomass (higher than 300 days) enable enrichment of slow growing microorganisms and forces biomass to use unusual substrates (e.g., PFAS). Additionally, the reduction in organic matter and competing constituents by microbial activity can enhance the efficiency of subsequent adsorption processes in the granular activated carbon (GAC) column. Furthermore, combining the SBBGR with GAC-based hybrid adsorption can be a suitable solution to further enhance PFAS removal. Nevertheless, this hybrid process has never been tested for the treatment of emerging contaminants, such as PFAS compounds from landfill leachate.

Based on these considerations, the present study aimed to assess the performance of a hybrid treatment combining the SBBGR system as the main biological treatment, followed by dual media GAC adsorption, for removing PFAS compounds from landfill leachate. Specifically, the study investigates the contribution of SBBGR to PFAS attenuation and matrix conditioning and evaluates downstream adsorption performance in terms of compound-specific breakthrough behavior, bed volume thresholds, and mass balance. The findings aim to support the design and operational optimization of integrated biological–adsorptive systems for effective PFAS control in complex wastewater matrices.

## 2. Materials and Methods

### 2.1. Hybrid SBBGR–GAC Treatment Process Design

**Figure 1:**
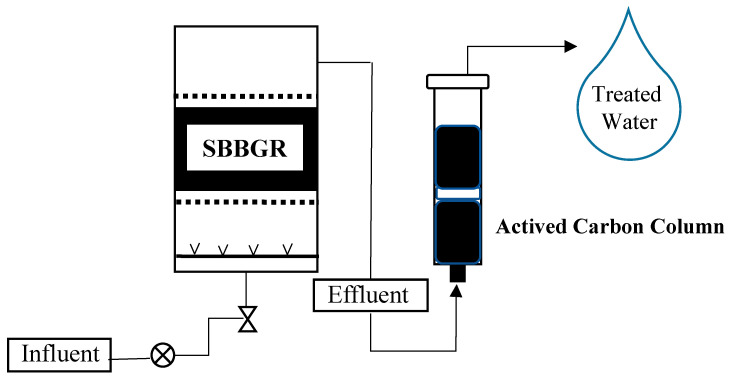
Schematic representation of the biological systems (SBBGR) coupled with a hybrid granular activated carbon polishing column.

The SBBGR setup comprised a cylindrical Plexiglas reactor (diameter: 19 cm; height: 100 cm; total volume: 28 L) partially filled with wheel-shaped Kaldnes plastic carriers (diameter: 8 mm; height: 7 mm; specific surface area: 690 m^2^/m^3^; porosity: 0.75), providing a fixed-bed volume of approximately 13 L within a working volume of 20 L, and operating under dynamic conditions comprising sequential anaerobic, anoxic, and aerobic phases (Figure 1). Aeration was provided with pure oxygen delivered via diffusers, and an external carbon source (sodium acetate) was added to sustain denitrification. The system was fully automated using a programmable logic controller (PLC) and included peristaltic pumps for influent feeding and internal recirculation. The sludge retention time (SRT) exceeded 300 days, promoting the selection of slow-growing microbial communities. More detailed information about the SBBGR system functioning can be found elsewhere [12].

The biologically treated effluent was further polished in a fixed-bed column sequentially packed with two distinct types of granular activated carbon (GAC): a mineral-based GAC and a vegetal-based GAC derived from coconut shells. The activated carbons supplied by Erica company (Cavenago di Brianza, Italy) are intended for water and liquid treatment applications; their characteristics are detailed in Table 1. The dual-media adsorption filter was designed to exploit the complementary characteristics of the two GACs. A mineral-based GAC was first used as a pre-polishing stage for the SBBGR effluent, aiming to preferentially retain larger organic molecules and thereby reduce competition for adsorption sites in the subsequent stage. In the second step, a coconut shell-based GAC, characterized by a more developed microporous structure, was employed to enhance the selective adsorption of PFAS compounds. This sequential configuration was intended to optimize adsorption performance by improving target contaminant accessibility and maximizing the efficiency of each carbon type. The vertical glass column was sequentially packed with 1 g of mineral-based GAC (particle size 12 × 40 mesh; apparent density 480 ± 20 kg m^−3^), a thin layer of inert glass wool, and 1 g of coconut-shell-based GAC (particle size 12 × 40 mesh; apparent density 500 kg m^−3^), resulting in a total bed volume of approximately 4 mL (Figure 1). The column system operated continuously for over 31 days. The influent flow rate was maintained at 0.06 mL/min using a Perkin Elmer Series 200 Micro Pump (PerkinElmer Inc., Shelton, CT, USA), yielding a residence time of approximately 66.7 min (30 min per carbon layer). The maximum PFAS removal efficiency was observed during the initial 17 days, with subsequent performance decline attributed to sorbent saturation.

### 2.2. Leachate Origin and Treatment Efficiency Evaluation

Leachate samples were collected from a non-hazardous landfill site in the Lombardy region of northern Italy. The identified perfluoroalkyl and polyfluoroalkyl substances (PFAS), together with their corresponding extended identification information, are summarized in Table 2.

### 2.3. PFAS Analysis

The calibration samples were prepared by using a standard PFAS mix purchased from Cambridge Isotope Laboratories (Tewksbury, MA, USA). The samples were collected daily in 90 mL aliquots using pre-cleaned polypropylene bottles. A 1 mL subsample was analyzed directly by high-resolution mass spectrometry without filtration or concentration. Dilution was applied to samples that exceeded the quantification range due to breakthrough on the adsorbent.

Quantitative analysis of PFAS was performed using ultra-high-performance liquid chromatography coupled to quadrupole time-of-flight mass spectrometry (UHPLC-QTOF-MS). The system comprised an Agilent Zorbax Eclipse Plus C18 analytical column (150 mm × 2.1 mm, 1.8 µm) and a matching guard column (Agilent Technologies, Santa Clara, CA, USA). Mobile phases consisted of (A) LC-MS-grade water containing 10 mM ammonium acetate and (B) LC-MS-grade methanol (Carlo Erba Reagents, Val-de-Reuil, France). The flow rate was 0.3 mL/min, and the oven temperature was maintained at 40 °C. The elution gradient was programmed as follows: 0–2 min, 5% B; 2–4 min, linear increase to 70% B; 4–9 min, linear increase to 100% B; 9–14 min, 100% B; 14–14.5 min, return to 5% B; 14.5–20 min, re-equilibration. The injection volume was set to 100 µL.

Mass spectrometric detection was conducted using a SCIEX TripleTOF 5600+ system operated in negative electrospray ionization (ESI–) mode (SCIEX, Framingham, MA, USA). The ion source parameters were set as follows: curtain gas, 25 psi; collision gas, 9 psi; ion spray voltage, −4500 V; source temperature, 400 °C; gas 1 and gas 2, 50 psi; declustering potential, −80 V; collision energy, −50 V; collision cell exit potential, −13 V. Data acquisition and processing were performed using SCIEX Analyst^®^ OS software (enbadded instrument software).

### 2.4. Instrument Calibration and Quantification

PFAS concentrations were quantified using external calibration against a certified reference standard (Wellington Laboratories, Canada) comprising eight target analytes at 50 µg/mL and two at 100 µg/mL. A working stock solution (10 ng/mL) was prepared in methanol and further diluted in LC-MS grade water to generate calibration levels of 1, 5, 10, 20, 50, 100, and 200 ng/L. Each standard and sample was spiked with 10 µL of an isotopically labeled internal standard mixture (EPA IS Mix, 10 ng/mL). Calibration curves were evaluated for linearity (R^2^ > 0.995), and recovery was assessed via replicate injections.

### 2.5. Breakthrough Calculations and Data Processing

Breakthrough performance was evaluated using normalized concentrations (C/C_0_), where C is the effluent concentration at time t, and C_0_ is the corresponding influent concentration. Breakthrough thresholds of the bed volumes at 10% and 50% were expressed as BV_10_ and BV_50_, defined as the number of treated bed volumes at which effluent concentrations reached 10% and 50% of influent levels, respectively. The bed volume (BV) thresholds were obtained by linear interpolation between adjacent C/C_0_ data points. Measurements failing quality assurance/quality control (QA/QC) criteria or missing values were treated as not available (NA), and no extrapolation was applied beyond the last valid observation. Analytes monitored over shorter operational windows (e.g., PFHpA and 6:2 FTSA) were evaluated only within their respective sampling periods and excluded from comparative BV or mass-balance analyses.

### 2.6. Mass Balance Calculations

Integrated mass balances were calculated to quantify PFAS removal across the dual media adsorption column. For each analyte, the inlet mass (Min), outlet mass (Mout), captured mass, and capture efficiency (%) were estimated by numerical integration of the concentration profiles over the treated volume, using measured influent concentrations and normalized breakthrough data. Integrations were performed over the common monitoring window to ensure comparability among compounds. Analytes monitored over shorter periods were excluded from cumulative calculations. Missing or QA/QC-failed measurements were treated as not available (NA) without extrapolation.

## 3. Results and Discussion

### 3.1. SBBGR Process Performance

Table 3 presents the average PFAS concentrations at the inlet and outlet of the SBBGR, along with the relative removal efficiency. PFOA, PFBS, PFOS, and PFBA exhibit the highest concentrations, reaching up to 281,300, 68,645, 22,670, and 13,235 ng L^−1^. Together, these four compounds account for 92% of the total concentration of PFAS considered (419,165 ng L^−1^), highlighting that their removal largely controls the performance of the SBBGR system. This observation aligns with Huang et al. [20], who found that such compounds as PFBS and PFBA often contributed over 40% of the total PFAS mass in municipal solid waste landfill leachate. After SBBGR treatment, the considered PFAS concentration (ΣPFAS_12_) decreased to 205,270 ng L^−1^, corresponding to an overall 51% reduction, which may be attributed to the particular structure and properties of the biomass (i.e., a mixture of biofilm and granules showing high hydrophobicity) and the dynamic conditions (arising from the sequential operation) which enable absorption of hydrophobic micropollutants on the biomass. The latter operates at hydraulic residence times greater than a year with concrete possibilities of potential PFAS destruction. Furthermore, compound-specific responses were heterogeneous: PFHpA (93%), PFNA (73%), PFHxS (67%), PFOS (57%), and PFOA (56%) declined markedly due to the high hydrophobicity and long retention times of the SBBGR biomass, enabling hydrophobic substances (e.g., long-chain PFAS) to be adsorbed and degraded [32], whereas PFBA (12%) and 6:2 FTSA (19%) were only marginally reduced, which could be due to their less hydrophobic nature. These results are in good agreement with previously reported studies [12,19].

Unexpectedly, the concentration of PFPeA increased slightly after treatment (Table 3), consistent with biotransformation of precursors and/or desorption from biofilm carriers during biological processing—a phenomenon also observed at full-scale wastewater treatment plants (WWTPs) [33,34]. Mechanistically, the comparatively higher removals for long-chain PFAAs (e.g., PFNA, PFOS) align with their greater hydrophobicity and stronger sorption to biomass/sludge. In contrast, short-chain PFCAs (PFBA, PFPeA, PFHxA) exhibit lower sorption affinity and therefore persist in the aqueous phase [35]. Generally, the sorption mechanism also depends on headgroup: PFSAs typically adsorb more strongly than PFCAs of equivalent chain length, a trend consistently shown in column/isotherm studies and reflected in practice [34,36]. Consistent with these processes, recent sludge surveys report preferential partitioning of long-chain PFAS to sewage sludge, while short-chain species remain predominantly in the effluents, matching our SBBGR observations [37]. From a process-function perspective, biofilm-based reactors such as the SBBGR primarily condition the matrix by reducing organic matter and co-contaminants, and by stabilizing hydraulics, rather than mineralizing PFAS [38].

### 3.2. Breakthrough Behavior in the GAC Column

Breakthrough performance was evaluated for compounds monitored over a sufficiently stable operational window to allow robust BV_10_ and BV_50_ determination. Accordingly, Table 4 reports threshold-based metrics only for analytes for which BV values could be calculated within the monitored bed volume range, ensuring comparability across compounds.

**Figure 2:**
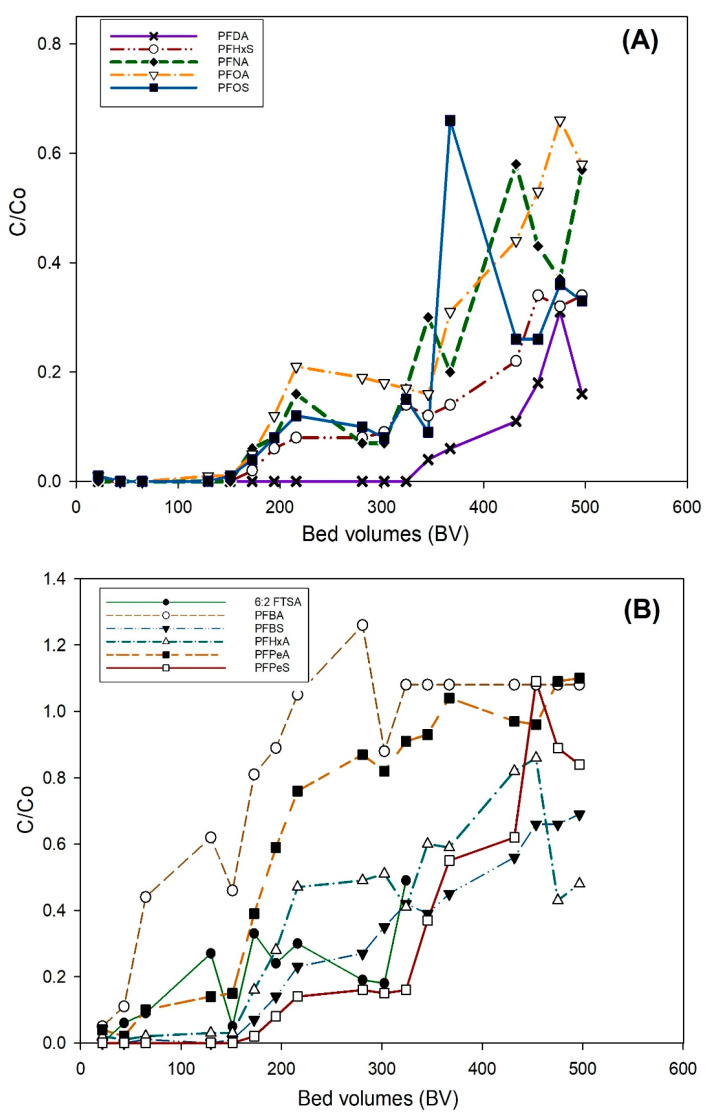
Normalized breakthrough curves (C/C_0_) of selected long- and medium-chain PFAS (**A**) and short-chain PFAS (**B**), plotted versus treated bed volumes (BV) for two fixed-bed column experiments.

Figure 2 illustrates the breakthrough behavior of PFAS compounds in the dual media carbon column, while Table 4 summarizes the PFAS breakthrough characteristics in the same system, reported as bed volumes at 10% and 50% breakthrough. The curve’s progressive breakthrough occurred with distinct, compound-dependent behavior. For example, short-chain PFCAs broke through first, followed by sulfonates of intermediate chain length. In contrast, long-chain PFCAs/PFSAs exhibited a delayed breakthrough and sustained low C/C_0_ values over an extended operating period (Figure 2). The latter exhibit an initial phase with negligible effluent concentrations (C/C_0_ ≈ 0), up to approximately 100–150 BV, indicating effective removal of the activated carbon during the initial stages of operation (Figure 2A). This trend is consistent with the idea that hydrophobic and electrostatic interactions govern PFAS adsorption, whereby an increase in perfluorinated carbon chain length enhances sorption to the medium [39,40]. In contrast, Figure 2B shows a substantially earlier breakthrough, with multiple analytes reaching C/C_0_ ≥ 0.5 before 300 BV and approaching full breakthrough (C/C_0_ ≈ 1) by approximately 450–500 BV (Figure 2B). This suggests reduced overall retention performance in the column configuration or influent condition. In addition, it is well known that the activated carbon properties (e.g., pore structure and distribution, surface specific area) also play a critical role in determining retention behavior [41]. Microporous materials (e.g., activated carbon) promote pore-filling mechanisms that preferentially stabilize longer-chain PFAS, while mesopores facilitate mass transfer but provide weaker confinement [42]. As these high-affinity micropores become saturated, adsorption shifts toward lower-energy external surfaces, resulting in accelerated breakthroughs, particularly for short-chain compounds [43]. Parallelly, Park et al. [44] reported that among positively charged activated carbons, materials with higher microporous surface areas showed greater apparent adsorption capacities for hydrophilic and moderately hydrophobic PFAS, while mesoporous carbon demonstrated progressively higher adsorption of more hydrophobic PFAS at later breakthrough stages compared with microporous carbon, likely as a result of reduced pore blockage. Overall, the results obtained are consistent with previous reports. For instance, Gagliano et al. [21] showed that GAC selectively removes long-chain perfluoroalkyl substances (PFAS), achieving almost complete adsorption of PFOA and PFOS, whereas short-chain species (e.g., PFBA and PFBS) exhibit rapid breakthroughs due to their reduced hydrophobicity and competitive displacement by longer-chain compounds. Likewise, comparative studies of sorption media consistently show weaker affinities for short-chain PFAS, highlighting the persistent challenges associated with their effective retention El Barkaoui et al. [19]. Notably, breakthrough curves display non-monotonic fluctuations, particularly at intermediate bed volumes. These variations likely reflect influent concentration variability, analytical uncertainty, and dynamic mass-transfer processes within the column. Therefore, BV_10_ and BV_50_ values were determined by linear interpolation between adjacent data points, as described in the Section 2, to provide standardized performance metrics.

Furthermore, Table 4 quantitatively confirms the breakthrough trends observed in Figure 2, revealing preferential retention of longer-chain PFAS and rapid breakthrough of short-chain species. For example, PFBA reached BV_50_ at ~88 bed volumes (~4 days), while PFOA and PFOS reached BV_50_ only after ~446 and ~361 bed volumes (~21 and ~17 days), respectively. PFHxS and PFDA did not reach BV_50_ within the monitored window, consistent with increasing hydrophobicity and pore-filling interactions with perfluorinated chain length, as well as the generally stronger sorption affinity of PFSAs relative to PFCAs of equivalent carbon number. Consequently, short-chain PFCAs exhibit lower loading capacities and earlier breakthroughs, whereas long-chain PFAS remain effectively retained for longer periods. This affinity ranking under a real effluent matrix agrees with controlled column studies and recent syntheses of PFAS adsorption performance [36]. From an operational perspective, the BV-ordered hierarchy (PFBA < PFPeA < PFPeS ≈ PFBS < PFOS < PFOA, with PFHxS and PFDA exceeding the BV_50_ window) provides a practical framework for column management. In particular, compliance is governed primarily by most mobile short-chain PFCAs, suggesting that increasing empty bed contact time (EBCT), implementing dual-media configurations such as GAC and anion exchange (AIX), or scheduling media replacement based on early BV thresholds may be necessary to maintain low effluent concentrations across the PFAS spectrum.

### 3.3. Mass Balance of the GAC Column

**Figure 3:**
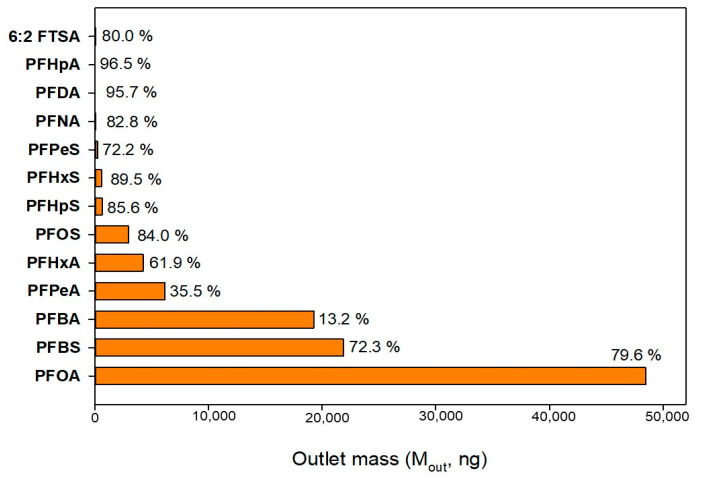
Cumulative PFAS outlet mass (Mout) and corresponding capture efficiencies of the GAC column.

Table 5 summarizes the outlet mass and capture efficiency of individual PFAS compounds across the GAC column, providing insight into compound-specific breakthrough behavior and overall treatment performance. Cumulative mass integration over the monitored runtime was used to quantify total PFAS capture at the system level. This mass-based approach incorporates all analytes with sufficient concentration–time data for numerical integration, thereby extending performance evaluation beyond BV_50_-based comparisons. Interestingly, the results illustrate that across the full operational window (~1.9 L treated; ~500 bed volumes), the dual media column captured approximately 73% of the total influent PFAS mass. Furthermore, as shown in Figure 3 and summarized in Table 5, the cumulative outlet masses and capture efficiencies clearly demonstrate the dual media system’s selective adsorption behavior toward individual PFAS. For example, PFOA dominated the influent load (~61% of the total mass) and, despite strong retention (~80% capture), remained the largest contributor to the effluent mass, followed by PFBS and PFBA. In contrast, long-chain PFAS such as PFHxS, PFOS, PFNA, and PFDA exhibited consistently high capture efficiencies (>80–95%), whereas short-chain PFCAs (PFBA and PFPeA) showed substantially lower retention (<40%), reflecting their earlier breakthrough and higher mobility. These mass-based trends are consistent with the affinity hierarchy derived from breakthrough analysis (Section 3.2), confirming that sorption strength increases with perfluorinated chain length and is generally higher for sulfonates than for carboxylates. Consequently, although long-chain PFAS are preferentially retained, short-chain species disproportionately contribute to the residual discharge.

## 4. Conclusions

The combined SBBGR–GAC treatment train demonstrated selective but incomplete removal of PFAS, governed primarily by chain length and functional group chemistry. The SBBGR achieved a moderate overall reduction (≈51% of ΣPFAS_12_), driven largely by effective attenuation of long-chain PFAAs and PFSAs (e.g., PFHpA, PFNA, PFHxS, PFOS, and PFOA), consistent with their higher hydrophobicity and stronger sorption to biofilm biomass. In contrast, short-chain PFCAs (notably PFBA and PFPeA) exhibited poor removal, with PFPeA even showing net formation, likely due to precursor biotransformation and/or desorption processes. These results confirm that the SBBGR primarily conditions the wastewater matrix, preferentially transferring long-chain species to solids while leaving mobile short-chain compounds in the aqueous phase.

Subsequent treatment with the dual media GAC column substantially enhanced PFAS control, achieving ~73% capture of the total influent PFAS mass over ~500 bed volumes. Breakthrough analysis revealed a clear affinity hierarchy, with rapid breakthrough of short-chain PFCAs (PFBA BV_50_ ≈ 88) and delayed breakthrough or sustained retention of long-chain PFAS (e.g., PFOA BV_50_ ≈ 446; PFOS BV_50_ ≈ 361; PFHxS and PFDA not reaching BV_50_). Mass balance results corroborated these trends, showing consistently high capture efficiencies (>80–95%) for long-chain PFAS, while short-chain species exhibited substantially lower retention (<40%), thereby dominating residual effluent loads.

Overall, the integrated system effectively mitigates long-chain PFAS but remains constrained by the early breakthrough and poor sorption of short-chain compounds, which ultimately control compliance and operational lifespan.

From an operational perspective, combining biological pre-treatment with downstream adsorption provided complementary removal mechanisms: the SBBGR reduced overall PFAS loading and matrix complexity, thereby enhancing carbon performance, while the dual media column supplied the polishing step required to achieve substantial mass removal. Compared with conventional WWTPs, where PFAS attenuation typically remains below 20%, the integrated system achieved markedly higher overall capture (~73%). These findings highlight that while biological pretreatment improves downstream GAC performance by reducing matrix interferences, robust management of short-chain PFAS will require additional strategies, such as increased EBCT, staged or dual-media configurations (e.g., GAC coupled with anion exchange), and replacement schedules based on early breakthrough thresholds. Collectively, this study underscores both the strengths and limitations of conventional biological–adsorptive treatment trains and emphasizes the need for targeted process optimization to achieve comprehensive PFAS removal across the full molecular spectrum.

Future studies should expand the analytical scope to include ultrashort-chain PFAS such as trifluoroacetic acid (TFA) in order to better assess transformation pathways and achieve a more complete mass balance in landfill leachate treatment systems.

## Figures and Tables

**Table 1:** Physicochemical characteristics of the granular activated carbons used in this study.

Parameter (Unit)	Standard/Method	Mineral-Based GAC	Coconut Shell-Based GAC
Product type	–	GAC	GAC
Raw material	–	Mineral-based	Coconut shell
Activation method	–	Physical	Steam (physical)
Main application	–	Water/liquid treatment	Water/liquid treatment
Iodine number (mg g^−1^)	ASTM 4607 [22]	≥950 (1000 typical)	1100
BET surface area (m^2^ g^−1^)	ASTM 3663 [23]	1100	1250
Pore size (nm)	–	10–50	<2
Particle size (mesh)	ASTM 2862 [24]	12 × 40	12 × 40
Particle size range (mm)	ASTM 2862 [24]	0.425–1.7	–
Fraction > 12 mesh (%)	ASTM 2862 [24]	≤5	5
Fraction 12–40 mesh (%)	ASTM 2862 [24]	≥90	–
Fraction < 40 mesh (%)	ASTM 2862 [24]	≤5	5
Apparent density (kg m^−3^)	ASTM 2854 [25]	480 ± 20	500
Density after drainage (kg m^−3^)	–	430 ± 20	450
Moisture content (%)	ASTM 2867 [26]	≤2	5
Hardness (%)	ASTM 3802 [27]	95	99
Ash content (%)	ASTM 2866 [28]	10	3
pH	ASTM 3838 [29]	Alkaline	Alkaline
Thermal reactivation	–	Possible	Possible
Standard compliance	–	UNI ISO EN 12915 [30]	UNI ISO EN 12915-1:2004 [31]

**Table 2:** Identified perfluoroalkyl and polyfluoroalkyl substances (PFAS) and their corresponding extended identification information in landfill leachate samples.

Compounds	Abbreviation	Chemical Formula	Limit of Quantification (ppt)
Perfluoropentanoic acid	PFPeA	C_5_HF_9_O_2_	70
Perfluorobutanoic acid	PFBA	C_4_HF_7_O_2_	49
Perfluorohexanoic acid	PFHxA	C_6_HF_11_O_2_	24
Perfluorooctanoic acid	PFOA	C_8_HF_15_O_2_	33
Perfluoroheptanoic acid	PFHpA	C_7_HF_13_O_2_	49
Perfluorononanoic acid	PFNA	C_9_HF_17_O_2_	13
Perfluoroundecanoic acid	PFUdA	C_11_HF_21_O_2_	20
Perfluorodecanoic acid	PFDA	C_10_HF_19_O_2_	34
Perfluorododecanoic acid	PFDoDA	C_12_HF_23_O_2_	36
Perfluorohexanesulfonic acid	PFHxS	C_6_HF_13_O_3_S	20
Perfluorobutanesulfonic acid	PFBS	C_4_HF_9_O_3_S	41
Perfluorooctanesulfonic acid	PFOS	C_8_HF_17_O_3_S	29
6:2 Fluorotelomer sulfonic acid	6:2 FTSA	C_8_H_5_F_13_O_3_S	28

**Table 3:** PFAS concentrations (mean value ± standard deviation) at the inlet and outlet of the SBBGR system, along with the corresponding removal efficiencies (%).

Compound	Influent (ng L^−1^)	Effluent (ng L^−1^)	Removal (%)
PFBA	13,235 ± 1138	11,682 ± 2920	12
PFPeA	4840 ± 71	5048 ± 1151	−4
PFHxA	8748 ± 384	5891 ± 1535	33
PFHpA	2711 ± 221	194 ± 595	93
PFOA	281,300 ± 99,136	124,800 ± 16,040	56
PFNA	608 ± 20	163 ± 22	73
PFDA	665 ± 147	349 ± 81	48
PFBS	68,645 ± 7064	41,655 ± 9629	39
PFHxS	8917 ± 1645	2951 ± 1992	67
PFHpS	6385 ± 2507	2338 ± 851	63
PFOS	22,670 ± 9857	9847 ± 1445	57
6:2 FTSA	438 ± 423	353 ± 92	19
ΣPFAS_12_	419,161	205,270	51

Negative values denote net increases.

**Table 4:** Breakthrough characteristics of selected PFAS compounds in the dual-media granular activated carbon column, expressed as bed volumes at 10% (BV_10_) and 50% (BV_50_) breakthrough (EBCT ≈ 66.7 min).

Analyte	Bed Volumes at 10% BV_10_	Bed Volumes at 50% BV_50_
PFBA	41	88
PFPeA	65	185
PFBS	–	397
PFPeS	–	361
PFOS	–	361
PFOA	–	446
PFHxS	n.r.	n.r.
PFDA	420	n.r.

n.r.: not reached within the monitored window.

**Table 5:** Cumulative Outlet mass (Mout) and capture efficiency (%) for individual PFAS derived from numerical mass integration over the monitored runtime of the GAC column.

Analyte	M_out_ (ng)	Capture (%)
PFBA	19,273.1	13.2
PFPeA	6188.9	35.5
PFHxA	4265.3	61.9
PFBS	21,899.9	72.3
PFPeS	248.5	72.2
PFOS	2994.7	84.0
PFOA	48,468.3	79.6
PFNA	53.3	82.8
PFDA	28.6	95.7
PFHxS	586.4	89.5
PFHpS	641.4	85.6
PFHpA	4.8	96.5
6:2 FTSA	85.6	80.0

## Data Availability

The original contributions presented in this study are included in the article. Further inquiries can be directed to the corresponding author.
